# Metabolites Alterations and Liver Injury in Hepatic Encephalopathy Models Evaluated by Use of 7T-MRI

**DOI:** 10.3390/metabo12050396

**Published:** 2022-04-27

**Authors:** Shigeyoshi Saito, Narumi Arihara, Reika Sawaya, Daisuke Morimoto-Ishikawa, Junpei Ueda

**Affiliations:** 1 Department of Medical Physics and Engineering, Area of Medical Imaging Technology and Science, Division of Health Sciences, Osaka University Graduate School of Medicine, Suita 565-0871, Japan; u478276a@ecs.osaka-u.ac.jp (N.A.); u010443b@ecs.osaka-u.ac.jp (R.S.); uedaj@sahs.med.osaka-u.ac.jp (J.U.); 2 Department of Advanced Medical Technologies, National Cerebral and Cardiovascular Center Research Institute, Suita 565-0871, Japan; 3 Radiology Center, Kindai University Hospital, Osakasayama 589-8511, Japan; morimoto-d@med.kindai.ac.jp

**Keywords:** hepatic encephalopathy, MR Spectroscopy, 7T-MRI

## Abstract

This study is to observe a thioacetamide (TAA) administered Hepatic encephalopathy (HE) model rats at three and ten days after TAA administration using liver MRI and brain MR Spectroscopy (MRS) by use of 7T-MRI. Forty-two Wistar rats (control group, *n* = 14) were intraperitoneally administered at 300 mg/kg (low-dose group, *n* = 14) or 400 mg/kg (high-dose group, *n* = 14) doses of TAA for induced of HE. At three days after TAA administration, glutamine (Gln) measured by MRS in high-dose and low-dose TAA groups showed significant increases in comparison to those of the control group (*p* < 0.05). Other metabolites measured by MRS showed no significant changes. Liver T_1ρ_ and T_2_ relaxation times significantly increased three days after TAA injection compared to pre-injection. There was a correlation between Gln levels in the brain and the relaxation time of the liver. Furthermore, Gln levels and relaxation time changed depending on the TAA dose. The Gln concentration in the brain increased with the deterioration of liver function, as inferred from the prolonged relaxation time of the liver. The prolonged relaxation time of the liver corresponded with the level of Gln in the brain. Gln concentration for the alterations of brain metabolites and T_1ρ_ relaxation time for the assessment of liver damage are useful markers for inter-organ association analysis in the HE model.

## 1. Introduction

Hepatic Encephalopathy (HE) refers to brain changes that occur in patients with advanced acute or chronic liver disease. As HE causes symptoms such as impaired consciousness and behavioral disturbances, it is categorized as a reversible neuropsychiatric syndrome that can range from early-stage cognitive deficit to coma [1]. HE is not a single clinical entity, and the pathophysiology resulting in brain dysfunction is not fully understood. Early-stage HE is difficult to diagnose because the detailed causative mechanisms have not yet been clarified [2]. Tools that can detect HE at an early stage may improve patient treatment and prevent poor outcomes.

In patients with HE, liver failure leads to hyperammonemia, an increase in blood ammonia levels. Previous studies have reported that ammonium (NH_4_^+^) and glutamine (Gln) are critically involved in HE pathogenesis; however, the molecular mechanisms leading to HE have not been fully elucidated [3]. Detoxification of NH_4_^+^ and other harmful substances is an important function of the liver, and when liver function declines, these substances easily accumulate in the brain. NH_4_^+^ can enter the brain and promotes Gln accumulation in astrocytes. Gln synthesized by astrocytes causes increased osmotic pressure in the brain, astrocyte swelling, and cytotoxic brain edema [4,5]. ^1^H magnetic resonance spectroscopy (MRS) is widely used to analyze metabolites such as Gln in detail noninvasively, especially in the brain. In magnetic resonance imaging (MRI) using low magnetic field strengths, such as 1.5 or 3.0 T, the metabolites, such as glutamine and glutamate, [Glu] overlap. ^1^H MRS measurements using high-field 7T-MRI characterized by its excellent spectral resolution provide information about alterations in neurotransmitters, oxidative stress, osmotic adjustment, and disturbances in cerebral metabolites [6] in more detail than MRS measurements using low-field MRI.

It is also important to diagnose the liver pathology underlying HE. Recently, measuring T_1ρ_ relaxation times has been utilized as a noninvasive diagnostic method for liver diseases in addition to conventional scanning techniques such as T_1_-weighted, T_2_-weighted, and diffusion-weighted imaging. T_1ρ_ measures the relaxation time in the apparent static magnetic field direction following irradiating magnetization with a spinlock pulse [7]. T_1ρ_ MRI is reportedly useful for measuring the time of slow-moving molecules in vivo [8,9]. Wang et al. showed that measurements of T_1ρ_ relaxation times facilitate the diagnosis of liver fibrosis in a bile duct ligation (BDL) model [10]. Moreover, T_2_ values obtained using T_2_ mapping can be used to evaluate the histological staging of liver fibrosis in chronic liver disease in both animal models and human patients [11]. Quantitative liver MRI has become an important diagnostic tool for the long-term observation of liver diseases.

Various animal models have been used in liver disease research. Thioacetamide (TAA) is widely used as a carcinogenic substance that affects nucleoprotein metabolism and the nuclear size of hepatic parenchymal cells around the central vein of the liver [12,13]. Regularly administered TAA is mainly used to create chronic hepatitis models, but some reports describe the administration of TAA to generate acute hepatitis models. TAA-induced liver fibrosis is similar to human liver fibrosis in terms of hemodynamic and metabolic changes [14].

It is important to diagnose liver pathology and brain metabolites underlying HE in order to demonstrate that liver failure leads to hyperammonemia. No previous studies reported simultaneous measurement of liver damage and brain metabolite changes in liver disease models. This study aimed to use brain 7T-MRI and MRS to examine HE model rats on days 3 and 10 after TAA administration. In addition, the T_1ρ_ and T_2_ relaxation times in the liver were measured using 7T-MRI. Blood and liver biopsy specimens were obtained simultaneously and analyzed. Assessing the relationships between liver function and brain metabolites may lead to a deeper understanding of inter-organ communication in HE.

## 2. Materials and Methods

### 2.1. Animal Preparation

All experimental protocols were approved by the Research Ethics Committee of Osaka University (Number: R02-05-0). All experimental procedures involving animals and their care were performed following the Osaka University Guidelines for Animal Experimentation and the National Institutes of Health Guide for the Care and Use of Laboratory Animals. Animal experiments were performed on 42 male Wistar rats (7 weeks old) purchased from Japan SLC (Hamamatsu, Japan). All rats were housed in a controlled vivarium environment (24 °C; 12-h:12-h light: dark cycle) and were fed a standard pellet diet with water ad libitum. To generate HE model rats, TAA was administered once at a dose of 300 mg/kg (low-dose group, *n* = 14) or 400 mg/kg (high-dose group, *n* = 14) to Wistar rats (control rats, *n* = 14). Each of the three groups was further divided for MRI (*n* = 9) and blood/histological (*n* = 5) assessments.

### 2.2. Magnetic Resonance Imaging

The MR images of rat livers were acquired using a horizontal 7T scanner (PharmaScan 70/16 US; Bruker BioSpin, Ettlingen, Germany) equipped with a 40-mm inner diameter coil for the brain and a 60-mm volume coil for liver imaging. To obtain the MR images, the rats were positioned in a stereotaxic frame with a mouth to prevent movement during acquisition [15]. The body temperature of the rats was maintained at 36.5 °C with regulated water flow and they were continuously monitored using a physiological monitoring system (SA Instruments Inc., Stony Brook, New York, NY, USA).

For liver imaging, T_1ρ_ images were acquired with fast spin-echo under respiratory gating with the following parameters: repetition time (TR) = 2500 ms; echo time (TE) = 30 ms; rapid acquisition with relaxation enhancement (RARE) factor = 8; spin-lock frequency = 1500 Hz; time of spinlock = 2, 12, 22, 32, 42 and 52 ms; slice thickness = 1 mm; field-of-view = 32 × 32 mm^2^; matrix size = 192 × 192; slice number = 1; slice orientation = transaxial; resolution = 167 × 167 μm; and scan time = 6 min. T_2_ images were acquired with RARE using the following parameters: TR = 2500 ms; TE = 9, 18, 27, 36, 45, 54, 63, 72, 81, 90, 99, 108, 117, 126, 135, 144, 153, 162, 171, 180, 189, 198, 207, 216, and 225 ms; RARE factor = 8; slice thickness = 1 mm; field-of-view = 32 × 32 mm^2^; matrix size = 160 × 160; slice number = 1; slice orientation = transaxial; resolution = 200 × 200 μm; and scan time = 6 min, 40 s. MR images were acquired before TAA injection and on days 3 and 10 after TAA injection.

First, cross-sectional images captured in three directions were obtained for MRS positioning. Next, images of the same resolution were used to accurately position a voxel of 8 × 4 × 2 mm^3^ in each of the two hippocampi. Magnetic field homogeneity was ascertained using the Bruker MAPSHIM shimming protocol, and good shimming between 6.8 and 10.8 Hz was achieved in the voxels. MRS was performed using a point-resolved spectroscopy sequence (TR/TE = 2500/20 ms, combined with variable power and optimized relaxation delay [VAPOR] water suppression). The metabolite spectra were acquired using 256 repetitions with VAPOR and 32 repetitions without VAPOR for a total scan time of 12 min. The metabolite concentrations were quantified using the basis set of the LCModel [15,16,17]. The metabolite concentrations, including those of NAA + NAAG, Cr + PCr, Lac, Cho, Glu, Gln, and Ins, were quantified using the basic setting of the LCModel [16].

### 2.3. Biochemical Analyses of Blood Samples

Anesthetized rats underwent thoracotomy and heart puncture to collect 3 mL of whole blood. Blood was collected in empty tubes and centrifuged at 805× *g* for 10 min. After centrifugation, serum was collected and sent to the Oriental Yeast Industry Co., Ltd. (Shiga, Kyoto, Japan) for measurements of four substances in the blood: ALT, AST, ALP, and T-BIL. AST, ALT, and ALP were determined using the JCCLS standard for Molecular Methods (L Type-Wako AST J2, L Type-Wako ALT J2, Type-Wako ALP J2, FUJIFILM Wako Chemicals, Osaka, Japan). T-BIL levels were determined by enzymatic analysis (Nescauto VL T-BIL, Alfresa Pharma Corporation, Osaka, Japan).

### 2.4. Histological Examinations

Liver damage was determined using H&E staining according to previously published protocols. After sacrificing the animals, the livers were removed, fixed in 4% phosphate-buffered formaldehyde, and embedded in paraffin. Paraffin-embedded livers were cut into 4-µm sections and mounted onto positively charged slides (Sapporo General Pathology Laboratory Co., Ltd., Sapporo, Japan) [18]. Slide images were obtained using a microscope (BZ-X810, Keyence, Osaka, Japan).

### 2.5. Statistical Analysis

MRS data and the blood biochemical parameters ALT, AST, ALP, and T-BIL are presented as the mean ± standard deviation. Differences were compared using the one-way ANOVA followed by Tukey’s post-hoc test to evaluate T_1ρ_ and T_2_ relaxation times, brain metabolite concentrations, and blood levels of the four biochemical substances within each model. The results of the biochemical analyses are described as the mean ± standard deviation. Differences were compared using the one-way ANOVA followed by Tukey’s post-hoc test to evaluate biomarker concentrations between the control group and each TAA injection model. Pearson’s correlation coefficients and correlation analysis were performed to compare the two groups (T_1ρ_ relaxation times vs. brain Gln levels, T_2_ relaxation times vs. brain Gln levels). All analyses were performed using Prism 9 software (GraphPad Software, San Diego, CA, USA). Statistical significance was set at *p* < 0.05.

## 3. Results

### 3.1. Animal Characteristics

The average body weight of the rats before TAA administration was 124.6 ± 2.5 g. In the low-dose group (300 mg/kg TAA), the average body weights were 112.4 ± 3.2 g and 121.8 ± 2.7 g on days 3 and 10 after administration, respectively. In the high-dose group (400 mg/kg TAA), the average body weights were 109.8 ± 4.1 g on day 3 and 120.6 ± 3.1 g on day 10 after administration. The body weights in the low-dose and high-dose groups were significantly decreased on day 3 in comparison to the control group (*p* < 0.01). There was no significant difference between the three groups on day 10.

### 3.2. Blood Tests

To validate the biochemical analysis of blood samples from TAA-treated rats, serum parameters including aspartate aminotransferase (AST), alanine aminotransferase (ALT), alkaline phosphatase (ALP), and total bilirubin (T-BIL) levels were analyzed (Table 1). The levels of AST, ALT, and T-BIL were significantly increased in TAA-administered groups compared to the control group three days after TAA injection (*p* < 0.001). Likewise, ALP levels in the low-dose group were significantly increased on day 3, whereas those in the high-dose group were also increased without reaching statistical significance.

### 3.3. MRI Images

**Figure 1:**
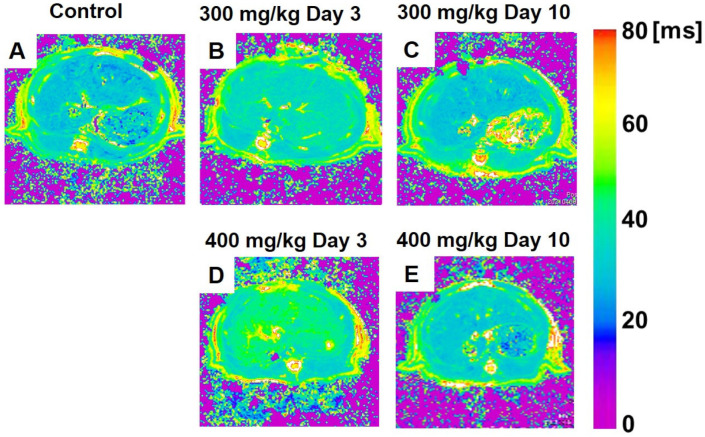
(**A**–**E**) Color-coded magnetic resonance liver T_1ρ_ maps showing representative results in the control (**A**), 300 mg/kg TAA injection (**B**,**C**), and 400 mg/kg TAA injection (**D**,**E**) models. TAA, thioacetamide.

**Figure 2:**
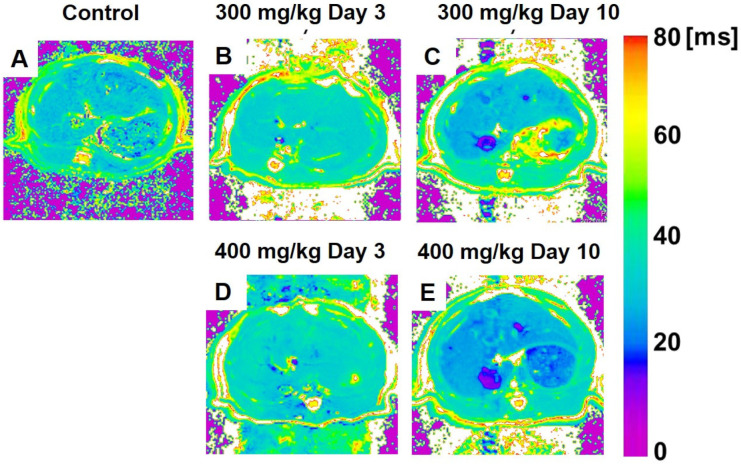
(**A**–**E**) Color-coded magnetic resonance liver T_2_ maps showing examples from animals of the control (**A**), 300 mg/kg TAA injection (**B**,**C**), and 400 mg/kg TAA injection (**D**,**E**) groups. TAA, thioacetamide.

Figure 1 shows the color-coded MR T_1ρ_ maps, and Figure 2 displays color-coded MR T_2_ maps, 300 mg/kg TAA-injected, and 400 mg/kg TAA-injected rats.

#### 3.3.1. T_1ρ_ Relaxation Time

**Figure 3:**
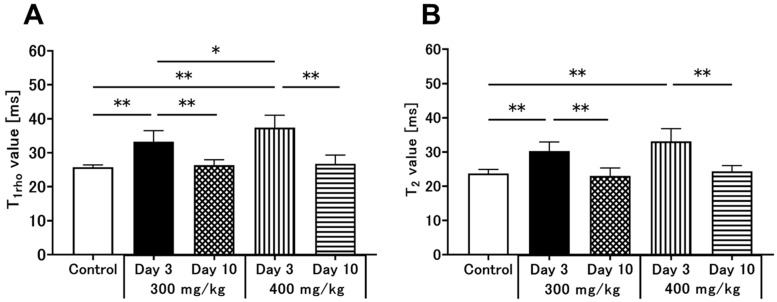
Changes in liver T_1ρ_ (**A**) and T_2_ (**B**) relaxation times in the high-dose (400 mg/kg) and low-dose (300 mg/kg) TAA groups compared to control. Data are presented as the mean ± standard deviation. Significance levels in comparison to control rats: * *p* < 0.05, ** *p* < 0.01. TAA, thioacetamide.

Figure 1 shows the color-coded MR T_1ρ_ maps in rats under control conditions or following 300 mg/kg or 400 mg/kg TAA injection. The statistical analysis of T_1ρ_ relaxation times is summarized in Figure 3A. The mean T_1ρ_ relaxation time of the liver in control animals was 25.7 ± 0.7 ms (Figure 1A and Figure 3A). In HE model rats, the relaxation times were significantly prolonged three days after TAA administration. In the low-dose group, the T_1ρ_ relaxation time was increased on day 3 (33.2 ± 3.3 ms, *p* < 0.01, Figure 1B and Figure 3A) but recovered on day 10 after TAA administration (26.3 ± 1.6 ms, Figure 1C and Figure 3A). In the high-dose group, the T_1ρ_ relaxation time was also significantly prolonged on day 3 (37.4 ± 3.6 ms, *p* < 0.01, Figure 1D and Figure 3A), recovering on day 10 after administration to control levels (26.8 ± 2.6 ms, Figure 1E and Figure 3A). Comparing the two TAA models, the high-dose group showed more prolonged T_1ρ_ relaxation times than the low-dose group. Three days after TAA administration, the T_1ρ_ relaxation time was significantly increased by 45% in the high-dose group in comparison to 29% in the low-dose group. Three days after the injection, there was a significant change because of the injection dose (Figure 3A, *p* < 0.05).

#### 3.3.2. T_2_ Relaxation Time

Figure 2 displays color-coded MR T_2_ maps from the control, 300 mg/kg TAA-injected, and 400 mg/kg TAA-injected rats. The statistical analysis of the T_2_ relaxation times is shown in Figure 3B. The mean T_2_ relaxation time in the liver was 23.7 ± 1.2 ms in the control animals (Figure 2A and Figure 3B). In the two HE models, the relaxation time was prolonged on day 3 after TAA administration. In the low-dose group, the T_2_ relaxation time was prolonged on day 3 (30.3 ± 2.7 ms, *p* < 0.01, Figure 2B and Figure 3B), but it recovered on day 10 after administration (23.0 ± 2.3 ms, Figure 2C and Figure 3B). In the high-dose group, this parameter was also significantly increased on day 3 (33.1 ± 3.7 ms, *p* < 0.01, Figure 3B, and this increase recovered by day 10 after TAA administration (24.3 ± 1.7 ms, Figure 3E and Figure 2B). Three days after TAA administration, the T_2_ relaxation times were significantly prolonged by 40% and 28% in the high-dose and low-dose groups, respectively (Figure 3B). No significant dose-dependent differences in T_2_ relaxation time were found for the two TAA models.

### 3.4. Brain MRS

**Figure 4:**
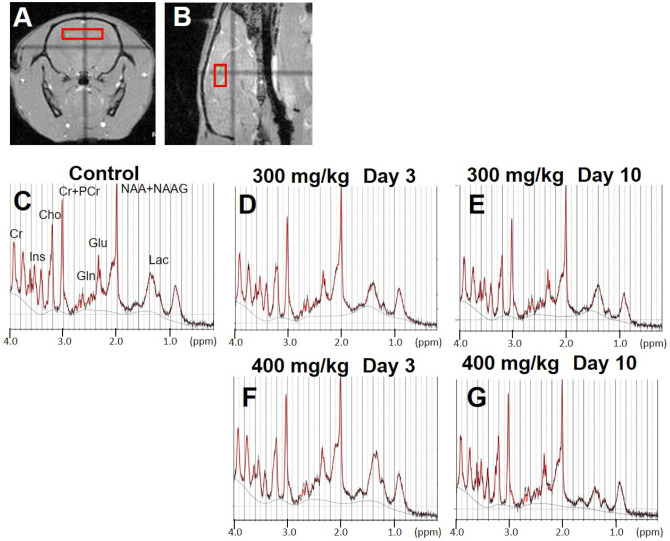
(**A,B**) show regions of interest in the hippocampus used for the analysis of MRS data. (**A**) is axial slice and (**B**) is sagittal slice sections. (**C**–**G**) indicate MR spectra compared to the control concentration. MRS, magnetic resonance spectroscopy. These five MR spectra show control (**C**), 300 mg/kg TAA injection (**D**,**E**), and 400 mg/kg TAA injection (**F**,**G**) groups. TAA, thioacetamide, NAA + NAAG, N-acetylaspartate + N-acetylaspartylglutamate; Cr + PCr, creatine + phosphocreatine; Cho, choline; Gln, glutamine; Glu, glutamate; Lac, lactate; Ins, myo-inositol.

**Figure 5:**
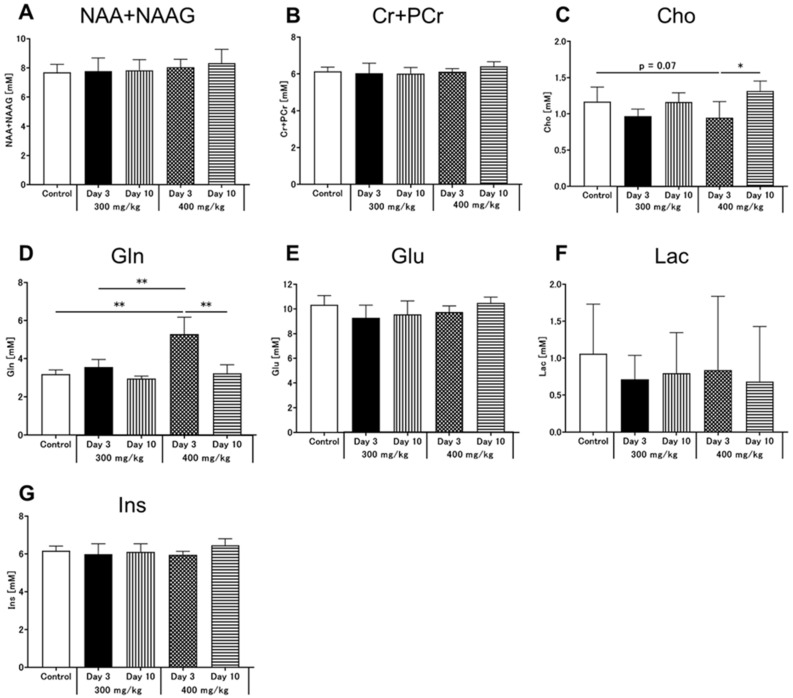
Results of the ^1^H MRS analyses. Data are presented as the mean ± standard deviation. Significance levels in comparison to control rats: * *p* < 0.05, ** *p* < 0.01. ^1^H MRS, proton magnetic resonance spectroscopy; (**A**) NAA + NAAG, N-acetylaspartate + N-acetylaspartylglutamate; (**B**) Cr + PCr, creatine + phosphocreatine; (**C**) Cho, choline; (**D**) Gln, glutamine; (**E**) Glu, glutamate; (**F**) Lac, lactate; (**G**) Ins, myo-inositol.

In the MRS images, regions of interest (ROI) were placed in the hippocampus to observe neurometabolic changes in brain MRS signals following TAA injection (Figure 4A,B). Figure 4C–G shows typical spectra of brain metabolite concentrations in all groups based on the LCModel method. Figure 5 shows the quantified concentrations of all analyzed brain metabolites (N-acetylaspartate [NAA] + N-acetylaspartylglutamate [NAAG], Cr + phosphocreatine [PCr], Cho, Gln, Glu, lactate [Lac], and Ins). Based on the control MR spectrum, only the high-dose group showed an increase in the Gln concentration after TAA injection (Figure 5D). The mean Gln concentration in the brains of control rats was 3.2 ± 0.2 mM. Compared to the control, Gln concentrations on day 3 after TAA administration were significantly increased to 5.3 ± 0.9 mM in the high-dose group (Figure 5D, *p* < 0.01). In the low-dose group, Gln concentrations were 3.6 ± 0.4 mM, slightly increased compared to the control rats, but there was no significant change (Figure 5D). At this time point, Gln concentrations significantly differed between the low-dose and high-dose groups (Figure 5D, *p* < 0.01). In addition, it significantly decreased ten days after administration compared to the concentration on day 3 in each group (Figure 5D, *p* < 0.01), and ten days after TAA administration, Gln levels were not significantly different between the two TAA groups. Furthermore, Cho levels were decreased three days after TAA administration in the high-dose group compared to those in the control group (Figure 5C, *p* = 0.07), and they significantly increased ten days after TAA administration (Figure 5C, *p* < 0.05). No significant differences in Cho levels were observed between the control and low-dose groups. All other metabolites also showed no significant changes.

**Figure 6:**
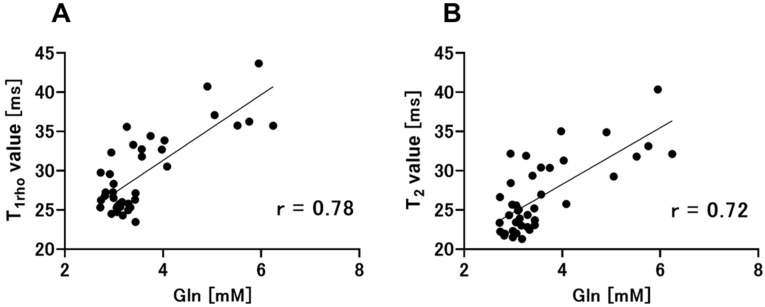
Pearson’s correlation coefficients and correlation analysis were performed to compare the two groups (T_1ρ_ relaxation times vs. brain Gln levels, T_2_ relaxation times vs. brain Gln levels). (**A**) The analysis of T_1ρ_ relaxation times and brain Gln levels also indicates a positive correlation between these parameters (r = 0.78, *p* < 0.01). (**B**) T_2_ relaxation times are positively correlated with brain Gln levels (r = 0.72, *p* < 0.01). Gln, glutamine.

Figure 6 shows the relationships of T_1ρ_ and T_2_ relaxation times with brain Gln levels. Figure 6A shows the positive correlation between T_1ρ_ relaxation time and brain Gln concentration (r = 0.78, *p* < 0.01). Figure 6B shows a positive correlation between T_2_ relaxation time and brain Gln levels (r = 0.72, *p* < 0.01).

### 3.5. Hepatocyte Stainings

**Figure 7:**
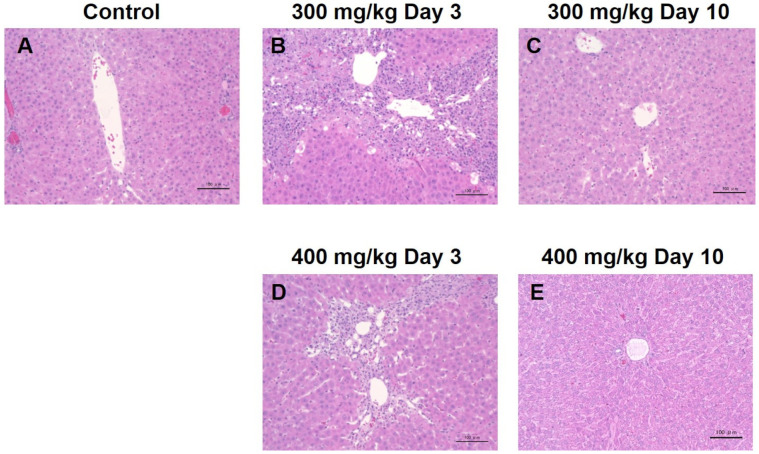
(**A**–**E**) show H&E stainings of the livers from control and TAA-injected rats. H&E, hematoxylin and eosin, TAA, thioacetamide.

Liver hematoxylin and eosin (H&E) tissue staining was performed before the TAA injection, as well as three and ten days after TAA administration (Figure 7). The normal control liver was densely populated by hepatocytes, and typical hepatocyte cords were observed. Three days after TAA administration, the liver showed a reduction in hepatocytic cellularity, particularly in the portal vein region, and infiltration of inflammatory cells was observed around the portal vein (Figure 7B,D). Ten days after TAA administration, the hepatocyte distribution recovered compared to three days after TAA administration (Figure 7C,E).

## 4. Discussion

Ammonia is a major neurotoxin implicated in human pathologies [19]. Gln, a by-product of ammonia metabolism, is capable of replicating many of the toxic effects of ammonia on neural cells. This is the first report of a simultaneous quantitative evaluation of Gln metabolites in the brain and T_1ρ_ and T_2_ relaxation times in the liver using 7T-MRI/MRS in a TAA-induced HE rat model. By simultaneously evaluating the effects of TAA on the brain and liver using high-field 7T-MRI, it was possible to evaluate the pathophysiology of this HE model that progresses in stages due to toxic ammonia effects and also provide basic data for the analysis of the brain–liver inter-organ communication.

### 4.1. Inter-Organ Communication between the Brain and the Liver

The brain plays a central role in metabolic regulation [20]. However, brain metabolism is not regulated by a single organ alone. The system is, among other factors, regulated by inter-organ communication between the brain and liver. In the HE model, on day 3 after TAA injection, Gln concentrations in the rat brain increased in a dose-dependent manner. In addition, dose-dependent increases in T_1ρ_ and T_2_ relaxation times in the liver were observed on day 3 following TAA administration. Ten days after TAA administration, Gln concentrations returned to control levels, as did the liver T_1ρ_ and T_2_ relaxation times in the low-dose group. These results show that Gln accumulation in the brain is closely related to T_1ρ_ relaxation times in the liver, especially three days after TAA administration. The measurements of T_1ρ_ and T_2_ relaxation times were similarly useful for evaluating a carbon tetrachloride (CCl_4_)-induced acute hepatitis model [18] and hepatic fibrosis in rats [21]. Furthermore, MRS was useful for evaluating the progression of HE in a BDL model [22,23]. In our study, metabolic changes in the blood and H&E stainings of the liver were also evaluated. These findings demonstrated that in the high-dose group, the degree of liver damage was more pronounced on day 3 but not on day 10 when the blood parameters had also returned to control levels. The prolonged relaxation times in the liver corresponded well to the increased Gln levels in the brain. These results indicate that the HE grade is influenced by the degree of liver damage. Therefore, MRS Gln and T_1ρ_ relaxation time measurements might be useful imaging methods to analyze inter-organ communication in HE models.

### 4.2. Early Neurometabolic Events in TAA-Induced Acute Liver Injury

Gln levels depended on the TAA dose and were significantly higher in the high-dose group than in the control group. The low-dose group also showed a slight increase in Gln levels. Moreover, Cho concentrations were decreased in both low- and high-dose groups three days after TAA injection. A previous study in a BDL model showed that Gln levels increased, whereas Cr, Gln, and Ins levels decreased with the progression of hepatic fibrosis [22,23]. The changes in Gln concentrations observed in the current study are consistent with those reported in previous studies. However, our study found changes in Cho levels that differ from those reported previously. Cho is a well-known precursor of acetylcholine and a cell membrane component commonly examined in MRS. It is a marker of cellular membrane turnover, and Cho levels are, therefore, elevated in neoplasms, demyelination, inflammation, and gliosis [24]. A decrease in choline levels was also observed in a previous study by Lanz et al. [25]. The Cho levels in our study suggest that the brains of TAA-administered rats were affected by neuroinflammation, thereby inducing alterations of Cho concentrations in the brain. The brain metabolites such as Cr, Gln, and Ins showed altered levels in BDL models of previous studies [22,23]. The Cr and Ins changed more than six weeks after BDL [22,23]. In the TAA-induced acute liver injury model, liver damage was milder and shorter than in the BDL model; therefore, alterations in brain metabolites may have been limited to Gln and Cho.

In the HE model of this study, the target structure of the brain MRS measurements was the hippocampus. In the hippocampus, short-term memory is thought to be formed by long-term potentiation, which increases the efficiency of neurotransmission upon stimulation and is presumed to be the basis of learning and memory [26]. Long-term potentiation is impaired in the hippocampus of rats affected by chronic hyperammonemia [27]. Hippocampal Gln levels in the acute hepatitis model showed changes similar to those in the chronic hepatitis model. HE is difficult to diagnose as dementia, possibly because hippocampus function is impaired by ammonia, affecting memory performance [28,29]. Therefore, this method using MRS brain and liver evaluations is expected to be applicable not only to HE models but also to other liver diseases that lead to brain disorders such as cirrhosis and fulminant hepatitis.

### 4.3. Early Alterations of T_1ρ_ and T_2_ Relaxation Times in TAA-Induced Acute Liver Injury

For the evaluation of liver damage in the acute hepatitis models, measurements of T_1ρ_ relaxation times might be more useful than those of T_2_ relaxation times. In this study, two doses of TAA were administered to induce acute hepatitis as a HE model in rats. Three days after TAA administration, dose-dependent differences in T_1ρ_ relaxation time were observed. By contrast, such dose-dependent changes in T_2_ relaxation times could not be observed. In a previous study, alterations in T_1ρ_ relaxation time were measured in a CCl_4_-induced acute hepatitis model [30]. In their study, T_1ρ_ relaxation times were also prolonged two days after drug administration; however, T_2_ relaxation times in the liver were not evaluated [30]. Several studies have described that T_1ρ_ measurements are useful for the noninvasive detection of liver fibrosis [31] and may not be affected by the presence of fatty liver [32]. The fibrosis degree was in these studies correlated with the degree of increase in T_1ρ_ relaxation times. Zhang et al. evaluated the values of T_2_, T_1ρ_, and diffusion metrics in the assessment of liver fibrosis in rats [33]. They concluded that for assessing liver fibrosis and monitoring disease severity, T_1ρ_ and diffusion-weighted imaging might serve as superior imaging biomarkers compared to T_2_ measurements [33]. For these reasons, T_1ρ_ relaxation times appear more useful parameters for the detection of acute liver damage than T_2_ relaxation times.

### 4.4. Limitations

This study has some limitations. First, additional types of rat hepatitis models exist that use different TAA doses, other types of drugs, or surgical BDL approaches. Farjam et al. reported blood biomarkers and liver tissue morphologies in three different models of acute hepatitis induced by varying doses of TAA [34]. In the current study, two different TAA doses were used. However, it might be useful to analyze the inter-organ correlation for a milder disease model using an even lower dose or a model with severer disease progression using a higher TAA dose to gain better insight into the effects of the liver disorder on the brain. Assuming there is a threshold of decreased hepatic function at which HE develops, it might be necessary to study this model with more detailed TAA dosage variations and time points and to increase the number of models.

Second, we consider using a chronic-phase model in the long term. In general, complications of liver diseases usually develop after 5–10 years, although it can take up to 30 years. TAA-induced hepatic fibrosis is similar to human hepatic fibrosis in terms of hemodynamic changes, as well as morphological and biochemical metabolic alterations. A chronic-phase model would be very useful for the study of human hepatic fibrosis [35] because the complications of TAA liver damage develop after a few days or weeks. Thus, long-term models are required to reflect liver disorders in humans.

Third, there were a few improvements in liver tissue stainings and blood tests. In this study, the group from which these MR data were obtained was not the group from which liver and blood samples were collected. Although a significant difference was not expected because similarly treated rats of the same type were used, it is most desirable to collect data from the same group. In addition, several previous studies measured NH_4_^+^ concentrations in the blood of chronic hepatitis model rats based on blood gas analyses [22,36]. In addition, it is available for the ammonia assay in serum and other biofluids using colorimetric assay kits. By measuring NH_4_^+^ concentrations or the concentration of ammonia in the blood, it is possible to investigate the effects of ammonia generated by liver injury in more depth.

Finally, the MRS data were obtained in previous studies by placing the ROI in the rat hippocampus to measure brain metabolite levels [25,29,37]. In the present study, data were also extracted from ROIs placed in the hippocampus. However, acquiring MRS data from the cortex or striatum instead of the hippocampus may reveal different brain metabolite changes in the same HE models.

## 5. Conclusions

Gln concentrations in the brain increase due to deteriorating liver function, as can be inferred from the prolonged relaxation time in the liver. The study results suggest that MRS-derived Gln concentrations as an indicator of alterations in brain metabolites and T_1ρ_ relaxation times for the assessment of liver damage are useful imaging parameters to analyze inter-organ communication in HE models.

## Figures and Tables

**Table 1:** Serum AST, ALT, ALP, and T-BIL levels of TAA-treated rats.

Treatment	AST (IU/L)	ALT (IU/L)	ALP (IU/L)	T-BIL (mg/dL)
**Control**	90.2 ± 11.4	33.2 ± 7.1	1347.8 ± 123.9	0.01 ± 0.00
**300 mg/kg TAA Inj Day 3**	675.8 ± 217.8 ***	393.5 ± 119.4 ***	2014.5 ± 191.0 ***	0.08 ± 0.02 ***
**300 mg/kg TAA Inj Day 10**	96.5 ± 7.5	47.0 ± 6.2	962.50 ± 46.9 *	0.02 ± 0.01
**400 mg/kg TAA Inj Day 3**	550.0 ± 237.8 ***	191.3 ± 82.4 **	1642.0 ± 355.1	0.09 ± 0.03 ***
**400 mg/kg TAA Inj Day 10**	112.5 ± 24.8	43.2 ± 7.6	1149.8 ± 85.5	0.02 ± 0.01

Significance levels in comparison to control rats: * *p* < 0.05, ** *p* < 0.01, and *** *p* < 0.001. ALP, alkaline phosphatase; ALT, alanine aminotransferase; AST, aspartate aminotransferase; TAA, thioacetamide; T-BIL, total bilirubin.

## Data Availability

The data presented in this study are available in the article.
